# Body Representation in Stroke Patients: A Systematic Review of Human Figure Graphic Representation

**DOI:** 10.3390/jcm14093098

**Published:** 2025-04-30

**Authors:** Olga Diyakonova, Valeria Habib, Marco Germanotta, Ksenija Taddei, Roberta Bruschetta, Giovanni Pioggia, Gennaro Tartarisco, Irene Giovanna Aprile

**Affiliations:** 1 IRCCS Fondazione Don Carlo Gnocchi ONLUS, 50143 Florence, Italy; odiyakonova@dongnocchi.it (O.D.); mgermanotta@dongnocchi.it (M.G.); ksenijataddei17@gmail.com (K.T.); iaprile@dongnocchi.it (I.G.A.); 2 National Research Council of Italy, Institute for Biomedical Research and Innovation, Via Leanza, Istituto Marino, 98164 Messina, Italy; roberta.bruschetta@irib.cnr.it (R.B.); giovanni.pioggia@cnr.it (G.P.); gennaro.tartarisco@cnr.it (G.T.)

**Keywords:** body representation, stroke, human figure drawing, Draw a Man Test, Draw a Person Test, self-perception, rehabilitation, cognitive dysfunction, mood disorders, activities of daily living

## Abstract

**Background:** Body representation is a complex process involving sensory, motor, and cognitive information. Frequently, it is disrupted after a stroke, impairing rehabilitation, emotional functioning, and daily functioning. The human figure graphic representation has emerged as a holistic tool to assess post-stroke outcomes. **Objectives:** This systematic review examines the methodologies of human figure representation tests and their application in assessing post-stroke body representation, emphasizing its role in bridging subjective patient experiences with objective metrics. **Methods:** This review follows the Preferred Reporting Items for Systematic Reviews and Meta-Analyses (PRISMA) statement. A literature search was conducted through the databases PubMed, Scopus, Embase, Web of Science, and Google Scholar, collecting publications eligible for qualitative analysis. We selected studies where patients drew human figures in the study design to assess body representation, involving exclusively the adult stroke population. The Newcastle–Ottawa Scale was used to assess the risk of bias. **Results:** Ten studies were analyzed. The tool demonstrated versatility in capturing unilateral spatial neglect, emotional disturbances, and functional independence. Qualitative metrics and quantitative indices correlated with cognitive deficits, mood disorders, and activities of daily living. Human figure representation also predicted rehabilitation outcomes, with improvements aligning with motor recovery. Innovations included digital quantification of evaluation metrics. **Conclusions:** Human figure graphic representation is a low-cost, adaptable tool bridging motor, cognitive, and emotional assessments in stroke survivors. While methodological variability persists, AI-driven analytics and standardized frameworks could enhance objectivity. Future research should prioritize validating parameters and developing hybrid models combining traditional qualitative insights with machine learning, thus advancing precision neurorehabilitation and personalized care.

## 1. Introduction

Stroke is listed as one of the leading causes of adult disability and ranks among the top three causes of death worldwide, impacting more than 15 million people each year [1,2,3]. Although stroke-related mortality in Europe is projected to decline by 17% from 2017 to 2047, its overall prevalence is expected to increase by 27% [4,5,6]. The condition significantly compromises survivors’ quality of life and imposes substantial economic and social impacts [7,8,9,10].

Neurological and long-term functional outcomes vary according to the extent and location of brain damage [11]. Physical and motor symptoms are present in approximately 80% of stroke cases. This symptomatology is often associated with deficits in several cognitive domains, such as mnestic, attentional, and language. Deficits in orientation and reasoning, dysphagia, and behavioral disorders can also be found [12,13].

After a stroke, the body representation, i.e., the way a person perceives and understands their own body, can be significantly altered. Body representation is a complex process involving several sources of information, top-down and bottom-up, such as visual, proprioceptive, and interoceptive. This information is continuously and constantly updated [14,15,16]. Precisely because of its complexity, several theoretical models have been proposed to theorize this construct. The dyadic and triadic taxonomies represent the two main theoretical models. The dyadic taxonomy distinguishes between body schema and body image [17,18,19,20]. Body schema includes the sensorimotor representations of the body that guide actions, while the body image includes all other representations of the body not aimed at action, whether perceptual, conceptual, or emotional (such as body perception, body concept, and body affect [18]. Several dissociations have been proposed in support of the dyadic taxonomy, such as the dual dissociation between deafferentation (body schema disruption) and numbsense (body image disruption) [20].

**Figure 1:**
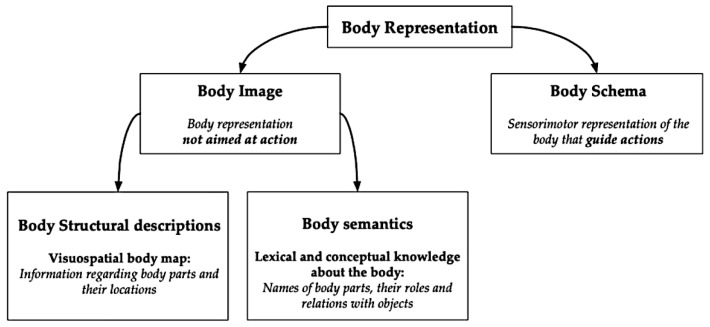
Body representation taxonomies. Original illustration created by the authors.

Building upon the dyadic framework, the triadic taxonomy was subsequently proposed. It includes body schema and body image, dividing body image further into two distinct components (Figure 1). Body structural descriptions (the visuospatial body map) consist of information regarding body parts and their locations. Body semantics (lexical and conceptual knowledge about the body) covers the lexis and conceptual content that concerns the body, that is, the names of the body parts, their roles, relations, and the interaction with objects [21,22,23].

Furthermore, both taxonomies concur on defining the body schema as a sensorimotor representation of the body, emphasizing the important connection between the body schema and action. Successful action cannot occur without an understanding of one’s bodily parameters, such as the size and strength of one’s limbs [24]. Among the components of body representation, the body schema appears to be the most impaired following a stroke, and in contrast to body image, which is lateralized in the left hemisphere, it does not seem to depend strictly on the activity of one brain hemisphere specifically. Moreover, deficits in body schema result in the parietal lobes or dorsolateral frontal cortex [21,25,26,27].

Following a stroke, people often experience profound changes with respect to the way they perceive their bodies. Such changes manifest in various clinical conditions, including asomatognosia, somatoparaphrenia, anosognosia for hemiplegia, and personal neglect. These conditions can be observed more frequently in the early stages after stroke and more often following right hemisphere lesions, but they can also occur after left hemisphere lesions and in chronic stroke [28]. A longitudinal study showed how disorders of body representation combined with visuospatial, attentional disorders, and hemiparesis can negatively impact the rehabilitation pathway [29] and, in particular, the recovery of subjects’ autonomies, such as dressing, hygiene, and functional mobility [25,30]. Picking up on the concept that body representation is a complex process involving several sources of information, it is important to point out that, in addition to somatosensory and neurological processes, emotional factors are also called into play. Such changes in the perception of one’s body necessarily require a process of acceptance and the creation of a new body image from an emotional as well as cognitive point of view [26,31].

Drawing the human figure is one of the oldest psychological testing techniques [32] and dates back to the Draw a Man Test (1926) by Florence L. Goodenough [33]. In its first version, the test was conceptualized as an intelligence test for children, based on the assumption that drawing complexity reflects cognitive level. In fact, the total score on the test can be compared with developmental norms, in order to estimate cognitive level [34,35,36]. Subsequently, in 1963, Dale B. Harris created an alternative version, called the Draw a Person test (DAPT), which placed greater emphasis on cognitive aspects in adulthood. This version required participants to draw a man, a woman, and a representation of the self [35,37]. Finally, in 1949, Karen Machover proposed the Human Figure Drawing (HFD) with the aim of investigating the personality and emotional aspects of adults and children [36] based on a symbolic interpretation of graphic drawing features such as figure size, placement, and omissions. In fact, the HFD, unlike the other instruments described, serves as a projective assessment tool [37,38]. More recently, modified versions of the test were proposed to also evaluate body schema in patients with neurological impairments [26]. Researchers have increasingly emphasized how graphic drawings of human figures are useful for neuropsychological assessments of patients with conditions such as stroke, traumatic brain injury, and neurodegenerative diseases like Alzheimer’s. Impairments in graphic skills are frequently observed in cases of cognitive dysfunction following brain injury, particularly when the parietal regions are affected [39,40]. DAPT or HFD, due to their nonverbal nature and in light of the ease of administration, could prove to be a useful tool for assessing and monitoring cognitive changes over time, even following rehabilitation treatment [41].

The aim of this review is to examine the use of human figure drawing to investigate body representation in patients after a stroke, both from a methodological and content perspective. The focus is to examine scientific evidence that supports the application of body image representation in clinical practice and stroke research.

## 2. Materials and Methods

### 2.1. Design

The systematic review protocol was conducted based on the Preferred Reporting Items for Systematic Reviews and Meta-Analysis (PRISMA) 2020 [42]. The PRISMA checklists are presented in the Supplementary Materials Content (PRISMA_checklist and PRISMA_abstract_checlist). Furthermore, we adopted the Rayyan.ai tool (Rayyan Systems, Cambridge, MA, USA), an AI-assisted tool that facilitated but did not replace manual screening [43,44].

### 2.2. Research Questions

The following questions guided our review:

1. How was the human figure drawing administered in adult patients after stroke from a methodological point of view?
2. What useful information can be extracted from the application of the graphic representation of human figure drawing to adult patients after stroke in relation to body representation?

### 2.3. Search Strategy and Inclusion Criteria

A systematic search for relevant publications was conducted by the authors between December 2024 and January 2025 across the following electronic databases: MEDLINE/PubMed, Scopus, Embase, and Web of Science. The included studies were published between 1971 and 2003. To ensure consistency and avoid the risk of misinterpretation, only English-language publications were included.

The keywords and the MeSH terms used to formulate the search strategy were relative to stroke, its manifestations, and the several ways in which the human figure graphic representation is called. The detailed search approach is presented in Table S1, available in the Supplementary Materials.

Moreover, a search on Google Scholar was performed to identify additional relevant studies. Specific queries were selected to ensure a relatively low number of resulting hits (see Supplementary Materials, Table S2, for the search strategy).

We included studies conducted on stroke patients (post-acute, subacute, or chronic stroke) using the human figure drawing in the study design directly linked to body representation, with exclusively adult participation (≥18 years old) and available in English. We excluded publications on the pediatric population (<18 years old) and those with full-text not available. Moreover, studies based on a pictorial representation of the human figure or pictorial self-portraits were excluded. Finally, we excluded meta-analyses, reviews, and case reports. In particular, case reports were excluded because they usually describe isolated clinical observations without standardized procedures or control conditions, making them unsuitable for the synthesis of consistent methodological or clinical evidence. Three researchers were involved independently in the extraction process.

### 2.4. Data Extraction

The results of the database searches were imported into Rayyan.ai to streamline the review process. The tool was used to automatically detect duplicates and to support the following screening phases conducted by reviewers. The duplicates detected were manually removed, and title and abstract screening were performed inside the tool to determine the remaining results. Relevant studies’ full-text papers were obtained and entered the full-text screening phase according to the inclusion criteria. The conflicts in the inclusion decision phase were resolved through discussion by the three reviewers jointly. If excluded, the reason for exclusion was documented. Reference lists of included studies were screened for potentially relevant studies.

The summary of the studies’ characteristics was exported to a Microsoft Excel sheet including the study titles, study year, authors, publication journal (and its Impact Factor at publication year, where available), study design, objectives of the study, participants (age, gender, and diagnosis), metrics used, and study outcomes.

### 2.5. Risk of Bias Assessment

The Newcastle–Ottawa Scale (NOS), recommended by the Cochrane Collaboration [45,46], was used to assess the risk of bias.

For the cohort studies included in the review, the original version of the NOS was used, which is based on three main domains: participant selection, group comparability, and outcome assessment.

For cross-sectional studies, the modified version of the NOS was adopted [47]. This version was adapted specifically for cross-sectional studies, retaining the three main domains and introducing specific criteria to address the peculiarities of this study design. The choice of this tool is justified by the need for a standardized and validated approach to analyze the risk of bias in studies that collect data at a single point in time, without follow-up.

The score ranges (presented in Table 1 below), adjusted for each type of scale, were used to classify the risk of bias into low, moderate, or high.

## 3. Results

### 3.1. Search Results and Screening

**Figure 2:**
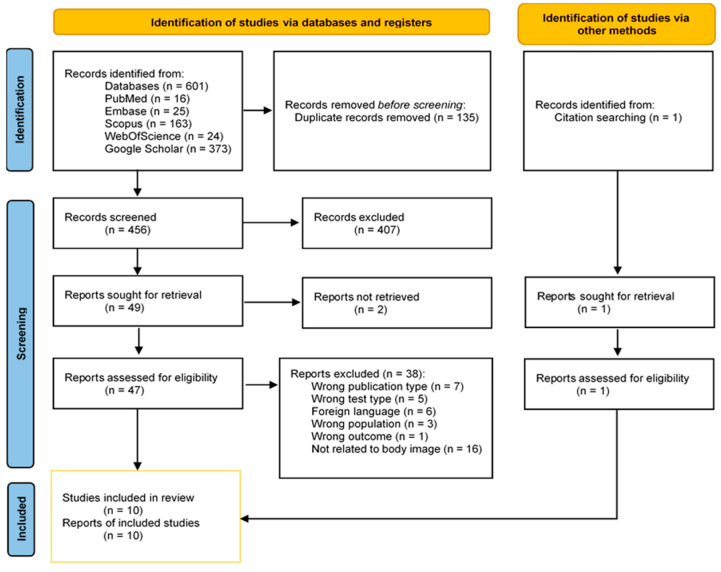
Search and selection of eligible articles [42,48].

The systematic research through search strings resulted in 601 study articles. The manual search produced one more result. Screening on title and abstract through Rayyan.ai was conducted on the studies resulting from a systematic search, thus on 601 studies. One hundred thirty-five duplicates were detected. The reviewers excluded 407 studies. Finally, 49 studies were screened for full-text assessment, 5 were excluded because of an inadequate publication type, 5 were using a different test for body image investigation, 6 were written in a foreign language, 3 did not include post-stroke patients, and 1 was excluded because of a different outcome type investigated. Ultimately, 10 study articles were included in the analysis. The PRISMA flow diagram is presented in Figure 2.

### 3.2. Study Characteristics

A total of 10 articles related to body representation in patients after stroke were reviewed [31,49,50,51,52,53,54,55,56,57].

The following information was retrieved from all articles and reported in Table 2: number of participants, gender, mean age, study objective, instrument used to measure body representation, other instruments used, and results. The mean age of the participants was 58.98 years, and most of the publications focused on middle-aged and older adults. Consistent with the research criteria, all studies reviewed focus on patients with acquired brain injuries. In just a few cases, the authors make a distinction with respect to the site of injury [49,50,51,52]. Only in one case [53] are patients divided by type of brain injury (ischemic or traumatic).

Body representation was assessed through basically four types of graphic tools: the Draw a Man Test [49,50,54], the Draw a Person Test [55], the Human Figure Drawing [31,51,53,56], or generically through self-portrait [52,57].

Regarding the aim of these studies, a first cluster is intended to create and refine drawing task-based tools to assess clinical aspects, such as the presence of neglect or body image distortion, potentially offering new diagnostic and predictive methods [50,51,56,57]. Two other studies investigate how alterations in spatial attention and body perception, following brain damage, can affect both self-representation (through drawings) and functional abilities [50,52]. Finally, a final cluster of studies explores the post-brain injury recovery pathway, highlighting how alterations in self-perception are closely related to psychological factors (mood, anxiety, depression) and the effectiveness of rehabilitation interventions [31,53,54,55].

To ensure better readability, an additional table summarizing the specific characteristics of the graphic human representation instrument has been reserved (Table 3). Specifically, in this table are the body representation assessment tool, clinical use of the tool, parameters used, delivery provided, type of assessment, and study topic. The various assessment tools for body representation have been used within the studies for different purposes. In our analysis work, we have identified: an assessment of patient clinical characteristics [31,51,52,53,56], a prognosis tool [49,57], and an instrument to monitor patient progression in the rehabilitation setting [54,55].

An additional insight that can be drawn from this collection of studies concerns the temporal distribution of the studies. The first studies examining the use of human figure representation in stroke patients were conducted in the 1970s. Based on our findings, and after considering the excluded papers, the interest in this topic has remained consistent to the present day. The trend observed in the 2020s highlights an increasing interest in utilizing recent technologies for drawing assessment, including the use of software-based automated tools [56] and the development of artificial intelligence algorithms (though not yet applied to stroke patients [58]). This growing interest appears to coincide with a new peak in the number of publications on the subject.

Through the analysis of these studies, we realized that there is a clear heterogeneity with respect to the parameters examined in the analysis of the drawings produced by the patients. It is also possible to divide these parameters into qualitative and quantitative. When we refer to qualitative parameters, we mean non-numerical data, not directly measurable, but that provide information regarding qualities or characteristics (for instance, the presence or absence of stylized or customized elements in the drawings or the intensity of the graphic trait). The presence or absence of specific items, however, if defined by objective and measurable parameters, may also be among the quantitative parameters. In fact, when we refer to quantitative parameters, we mean numerical and objective measurements collected through standardized measurements. Some examples are figure measurements (height or width of the figure), calculation of the ratio of different body parts, number of details included, symmetry, orientation of the figure, and the occupied area of the drawing. According to this classification, it is possible to divide the studies under investigation into two groups: those using qualitative parameters [49,50,52,54,55,57] and those using quantitative parameters [31,51,53,56]. Articles reporting both qualitative and quantitative parameters were therefore reported in the box corresponding to the type of parameters used predominantly. It is visible how the application of qualitative parameters prevails at the expense of quantitative ones. With respect to the temporal distribution, it can be seen that more recent studies tend to favor the use of quantitative parameters, while, in contrast, older studies tend more toward qualitative analysis.

Getting more specific with respect to the individual parameters examined in the different studies, it is possible to divide them into qualitative and quantitative as shown in Table 4.

Three categories of studies can be identified based on the instructions provided. The first group consists of studies that require drawing two human figures of opposite sexes [31,53]. The second group includes studies that ask for the production of a generic human figure [49,56]. Finally, the third group includes studies where participants are asked to draw themselves [51,52]. In some of the studies we reviewed, the instructions are not even explicitly stated [50,54,55].

**Figure 3:**
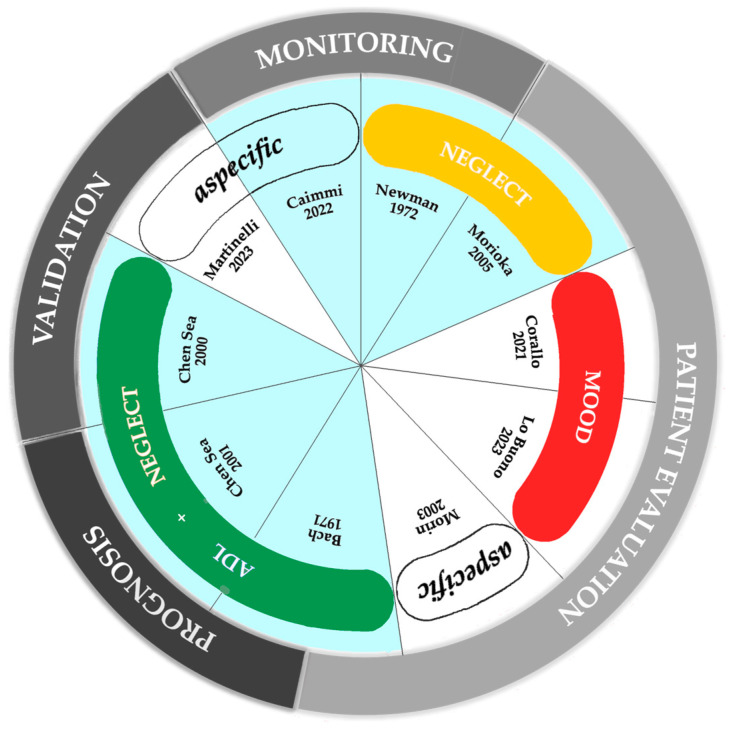
Topics and clinical use discussed in the articles reviewed [31,49,50,51,52,53,54,55,56,57]. The outer ring shows the clinical uses of the tool. In the inner ring, the topics are covered. Areas corresponding to studies applying qualitative parameters are shaded in light blue. In white are those applying quantitative parameters. Original illustration created by the authors.

To put it in a nutshell, as can be seen in Figure 3, the clinical applications for which this tool has been implemented range from monitoring the progression of recovery, both physical and cognitive, of the patient (in two articles [54,55]), prognosis regarding the success of their rehabilitation (in two other articles [50,57]), and evaluating specific aspects of their pathology (in five articles [31,51,52,53,57]). One study specifically focuses on the validation of the test’s use for diagnosing USN [49], while another recent article validates an innovative tool for quantitative assessment of drawing [56]. Additionally, in Figure 3, the areas corresponding to studies that employed a qualitative evaluation approach are shaded in light blue.

### 3.3. Study Quality

According to the Newcastle–Ottawa Scale (NOS), among the reviewed studies, seven exhibited low risk of bias [31,49,50,51,53,54,56], while three exhibited moderate risks of bias [52,55,57].

The comprehensive evaluation of each domain’s quality across the studies is illustrated in Table 5.

## 4. Discussion

Based on our literature review work, we can state that graphic representation of the human figure has been used in relation to very heterogeneous topics. In particular, the themes most frequently related to body representation are as follows: unilateral spatial neglect, activities of daily living, and mood disorders (Figure 3).

### 4.1. Body Representation and Unilateral Spatial Neglect

The area most extensively developed by the authors is the evaluation of the USN. Even within this theme, the tool has been used very heterogeneously. The fundamental assumption for which this tool has been applied to the USN is that individuals with an intact body representation tend to draw a human figure presenting homogeneous bilateral body parts, while patients with personal neglect (i.e., the inability to recognize or acknowledge the contralateral side of the body) tend to omit body parts contralateral to the brain injury, indicative of a disturbance in the perception and recognition of the body part. The Draw a Man Test was used to differentiate between personal and extrapersonal USN, as well as their impact on ADL [49,50]. In a second case, inspired by Bisiach and Luzzatti’s work [59] that demonstrated how patients with USN show difficulty in directing attention to the left portion of imagined visual scenes, self-portrait was used to infer body representation in patients with USN, This was accomplished by comparing self-portraits drawn with open versus closed eyes [52], with their results suggesting that body representation distortions also affect mental imagery.

The relationship between a complete human figure graphic representation and the outcome of a rehabilitation intervention in terms of recovery of ADLs has also been addressed. Bach and colleagues previously highlighted this connection in a broader context for stroke patients, even suggesting the use of this tool in a prognostic manner to assess the levels of autonomy and independence of hemiplegic patients following rehabilitation [57]. It is important to note that the authors do not explicitly specify what the test measures (as in the previous case, where the test was explicitly employed to assess neglect) but rather they associate the way the patient depicts themselves with various potential deficits caused by stroke and emphasize its connection to the recovery of ADLs. The idea is, in general, that a better represented drawing may signify a successful recovery. In this study, the prognosis, based on the correlation between patients’ autonomy after rehabilitation, and the way they present their self-portrait, yields an accuracy of 87%. Contextually, the authors propose several considerations regarding recurring drawing characteristics to be taken into account in this evaluation, based on clinical experience. Some examples are provided below:

– Omission of body parts suggests a denial of disability. For example, patients with severe aphasia may depict faces without mouths, or even completely empty faces. Hemiplegic patients frequently fail to represent arms or legs. Interestingly, it has been observed that wheelchair-bound patients who initially omit their legs in a drawing later depict them after regaining the ability to walk;
– Leaning or off-balance pictures suggest balance problems.

### 4.2. Body Representation and Mood Disorders

According to our review, another major theme studied using a graphic representation of the human figure is mood. Two of the studies we reviewed [31,53] agree on an association between mood disorders and body image distortions. HFD has been applied to explore implicit body representation (i.e., unconscious representation of the body that influences body-related perceptions, emotions, and behaviors), while the assessment of body image has been specifically delegated to the application of the Body Image Scale (BIS), a 10-item questionnaire investigating aspects of body image in ABI patients. Depression, in particular, has been found to impact body image, while anxiety is associated with both body image and implicit body representation, investigable through the use of the HFD. Unfortunately, the correlation between the two metrics has not been reported, but both have shown links to both depressive states and cognitive deficits. Lesion site plays a significant role in predicting body representation, with both anxiety and depression influencing these outcomes.

It is noteworthy that, in addition to mood assessment, Corallo and colleagues identified a relationship between body representation and cognitive functioning in patients with stroke. In particular, they highlight that performance in visual-spatial tests is a significant predictor of recorded performance in HFD [53]. This result is very understandable considering that HFD is a graphical test. Moreover, a further result emerges from the same study, whereby language performance (such as naming), abstraction performance, and orientation performance also result as predictors of performance on the HFD. These results highlight how this instrument draws afferent skills from multi-level cognitive domains and provides multiple levels of possible analysis. Consistent with this, although drawing a human figure may appear to be a simple and straightforward task, it actually requires a complex interplay between motor and cognitive skills.

### 4.3. Psychoanalytic Perspectives on Body Representation

Due to the complexity of the topic and the multiple levels of interpretation, body representation is a construct that has been analyzed from various perspectives, sometimes difficult to classify within any of the aforementioned topics. A significant example of multidisciplinary integration is provided by the study by Morin and colleagues [51], where self-portraits produced by stroke patients were analyzed with reference to the specular image, intended as the integration of body perception with the symbolic and imaginary components of identity. The specular image does not necessarily correspond to the physical or functional reality of the body but offers an idealized representation that supports psychic organization and self-perception as a subject. Through this study, the authors identified fragmentation of the specular image following both right and left brain lesions. Clusters were identified based on the characteristics exhibited in the drawings:

– Upright, complete, and clothed self-portraits were predominantly drawn by control participants;
– Upright self-portraits lacking clothing, hands, and/or mouth and eyes were found across all participant groups, predominantly among patients exhibiting speech disorders;
– Inclined self-portraits with unilateral omissions were predominantly drawn by patients with right brain lesions.

### 4.4. Clinical Applications of Body Representation Assessment Tools

We also find a high amount of heterogeneity with regard to the clinical use of the instrument. Our analysis identified the following main purposes of use: prognosis of patient autonomy at the end of treatment, evaluation of treatment response (i.e., outcome measure), and monitoring of rehabilitation progress over time (Figure 3). In addition, some studies focused on the validation of new uses of the tool. Chen-Sea and colleagues conducted an initial study to validate the use of drawing for identifying personal neglect. They addressed this issue by demonstrating good interrater reliability, although without comparing it to a gold standard [49]. In the subsequent study, the authors combine the Draw a Man Test with the Random Chinese Word Cancelation Test, emphasizing that the latter is not useful for identifying personal neglect, but only extrapersonal neglect [50]. Bach and colleagues suggest using self-portraiture to assess the relationship between the drawing and patient independence. Having found confirmation of this, the authors use the HFD for prognostic purposes [52]. In addition to being used as a prognostic tool, the graphic representation of the human figure has also been utilized as a tool for monitoring post-stroke recovery. Newman and colleagues used the Draw a Man Test to assess the evolution of body representation in hemiplegic patients, although with an unspecified scoring system and an observational interpretation of the quality of the drawing over time, primarily focusing on the deconstruction of the image or the actual omission of parts [54]. The patient’s recovery during treatment was assessed over time, on a weekly basis, using a specially designed evaluative scale that included the assessment of general motor function, sensory function, cognitive function, and language. Alongside improvements in these areas, interesting subsequent progress was observed in the results of the Draw a Man test, with examples of transitions from constructional apraxia to unilateral neglect, followed by a complete recovery of body image. These findings demonstrate a correlation between the evolution of the drawing and the patient’s overall condition.

**Figure 4:**
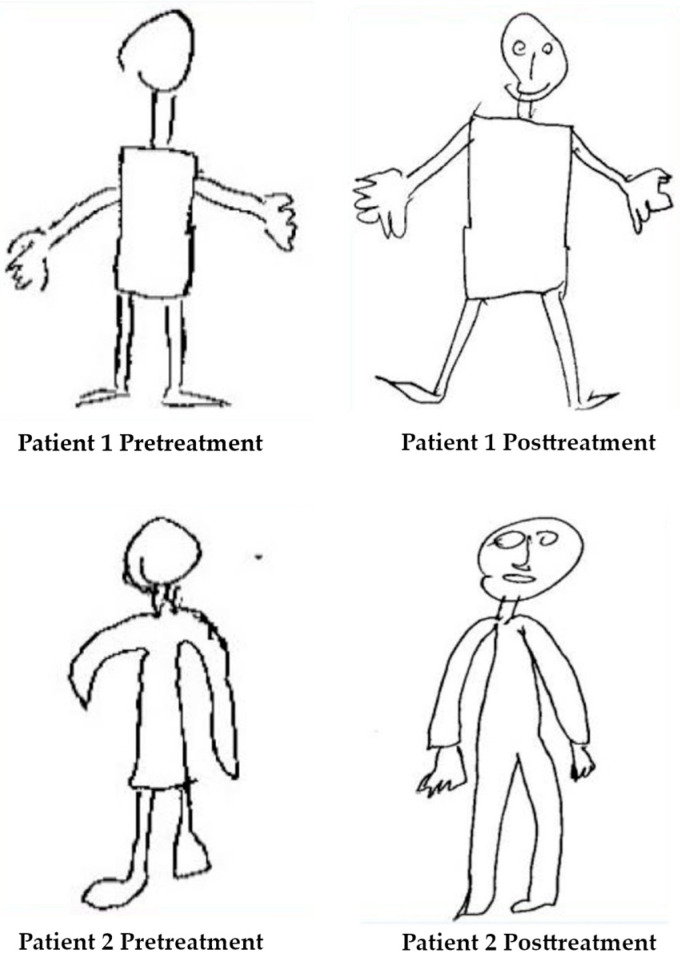
Pre- and post-treatment DAP test of two chronic patients. Adapted from Caimmi et al., 2022 [55]. Licensed under CC BY 4.0. Modifications were made to the original image.

In a more recent study [55], the Draw a Man Test was used to compare the mental body representation before and after robotic rehabilitation treatment in stroke patients, in order to assess the responsiveness to the treatment. Improvements were noted in the drawings produced by the patients (Figure 4).

Specifically, after the treatment, all body segments were drawn, respecting the correct proportions, and elements that were previously omitted, such as eyes, nose, and mouth, appeared. The authors interpret this result by hypothesizing that the robotic rehabilitation intervention may have increased brain connectivity between areas relevant to sensorimotor function, improving somatic awareness in these patients.

### 4.5. Methodological Considerations in Body Representation Tools

In addition to the topics and clinical uses of the tools, a third analysis focused on the parameters examined in the different applications of the graphical tool. In fact, to support the use of body image representation in clinical practice and stroke research, it is essential to understand how to interpret the graphic sign and according to which parameters to assign the scores. Our work revealed a heterogeneity in the types of parameters employed, suggesting that there is currently no established standard or clear preference for the use of either qualitative or quantitative measures. Some studies have favored qualitative parameters, while others have relied on quantitative measures, depending on the specific objectives of the study. Both approaches, in fact, present specific strengths and limitations. In general, it can be assumed that a qualitative interpretation can capture subjective meanings and dimensions related to body representation that may elude a purely quantitative interpretation and may be better suited to an in-depth study of the individual. On the other hand, studies that use a quantitative approach to analyze graphic representation may better support large-scale research or clinical settings and allow for an objective and replicable measurement of specific features that are better suited for comparison between patients or monitoring over time. In fact, while in the case of qualitative interpretations we rely on non-standardized guidelines that leave room for the practitioner’s interpretive skills, in the case of quantitative parameters we rely on standardized measurement tools or software that ensure greater uniformity and replicability. Moreover, by maintaining an approach that considers only the presence or absence of an element in the drawing, it is possible to obtain an evaluation that is not penalized by difficulties in using a non-dominant limb in certain cases or challenges in graphic production in others.

Clearly, a quantitative approach should be tailored to the specific goals of the analysis. In addressing the issue of spatial unilateral neglect, for instance, we noticed that none of the studies reviewed utilized a quantitative evaluation method. According to Chen-Sea’s 2000 study [49], the use of a score based on the overall completeness and accuracy of the drawing’s details lacks precision in capturing the spatial distribution of these elements. Consequently, the same score could correspond to very different graphic representations. For example, it would fail to capture the difference between a drawing that is generally incomplete and one in which exactly half of the body is missing relative to the sagittal axis. In this case, the author proposed a qualitative approach; however, a different quantitative approach investigating also the spatial features of the graphic representation of the human figure could be a better sign of the clinical condition to be investigated.

Interestingly, studies focusing on monitoring the rehabilitation process also use a qualitative approach. This is likely due to the fact that these studies—specifically those by Newman [54] and Caimmi [55]—did not treat body perception as a primary outcome for statistical analysis.

The studies utilizing quantitative assessment methods focus on evaluating the patient both for exploratory purposes, as in Morin et al. [51], and to identify the relationships between body image and mood disorders, as examined by Lo Buono and Corallo [31,53]. In these studies, the use of a score-based evaluation allowed for statistical comparisons between patient clusters and enabled a statistical correlation analysis between scales commonly employed in body dissatisfaction assessments and body perception. However, the quantitative evaluation process necessitates the involvement of a trained specialist and the allocation of time for detailed analysis. An innovative approach to facilitate this process was proposed by Martinelli et al., using manual image analysis software [56], with the potential to expand and refine the use of graphic representation of the human figure. An application, specifically developed (QDraw), allowed for a digital and standardized analysis of drawings and generated quantitative, numerical measurements of various body segments, which can be used with specific algorithms to calculate values of interest in the assessment of body representations. Compared to manual analysis, this approach allows for a significant reduction in time and minimizes the possibility of error in the coding of the graphic sign, promoting a standardization of the assessment. However, it remains anchored to the traditionally proposed metrics and still requires the operator’s intervention to support measurements.

The wide heterogeneity regarding the parameters used is also reflected by the different instructions given during the test administration. The lack of agreement between the different instructions likely reflects the different objectives underlying the various studies examined. A natural reflection arises: different words, by conveying different meanings, can guide the patients in various ways during the drawing production. Asking to draw a generic human figure, or oneself, specifying whether a full-body figure or not could lead to different graphic outcomes that are not entirely comparable with others. Interestingly, only three of the studies analyzed explicitly focused on a self-portrait request [51,52,57]. Specifically, two of these studies conducted exploratory work on self-perception, and in these cases, the choice of self-portraiture is more than justified [51,52,57]. All the remaining studies do not provide any commentary on the instructions given to the patients. Of course, what we are investigating is the possibility that body perception may be projected onto a generic human figure drawing. Nevertheless, where feasible, it seems reasonable to assume that requesting a self-portrait would be more appropriate for exploring body perception specifically.

### 4.6. Future Perspectives: Standardization, Innovation, and the Role of Artificial Intelligence

It is important to further emphasize that the studies identified are limited in scope, exhibit considerable heterogeneity, and are dispersed across an extended time frame, thus raising the question of the actual applicability of the tool in the proposed context. Nevertheless, the available evidence remains compelling, as it provides a multi-perspective analysis of a complex pathology and offers a simple tool for rehabilitation, despite the fact that in many instances it lacks support from quantifiable data or detailed statistical analysis. It appears prudent to advocate for the development of new metrics, grounded in detailed statistical correlations between the clinical characteristics of patients and the features of the drawings, which would preserve the depth of qualitative analysis while providing quantifiable and reproducible evaluations. While such an approach may have been unfeasible in the past, recent advancements in technology, particularly in artificial intelligence (AI) algorithms, now offer the opportunity to realize this potential. AI is assuming an increasingly central role across various productive sectors, including healthcare, offering promising opportunities to enhance patient care and overall quality of life [58,59].

Technologies that use AI do not simply replicate the manual analysis performed by the examiner but allow them to detect patterns or features that would otherwise be difficult to identify [60]. Some examples of technological applications might be the automated analysis of proportions against anatomical standards, the detection of features and pattern density, or finally, the training of AI models to recognize specific features of drawings associated with clinical conditions. The application of technologies brings advantages to greater standardization and objectivity in analyses, reducing the risk of bias, and greater efficiency due to the ability to process large masses of data. However, the future in this regard may lie in a hybrid model that integrates the application of new technologies with an interpretation that considers individual and subjective variables.

A significant challenge in the analysis of human figure drawings in post-stroke patients is the transition from qualitative clinical interpretations to objective, quantifiable metrics suitable for AI-based analysis. The proposed Table 6 provides a structured conversion framework, bridging traditional clinical assessment methods with computational approaches.

The proposed conversion framework systematically maps qualitative aspects (e.g., presence of eyes, hands, or clothing; structural disorganization; hemineglect) to measurable AI-compatible parameters such as feature completeness scores, symmetry indices, and spatial distribution metrics. Additionally, established quantitative metrics, including limb length, head size, axis of symmetry, and overall figure proportions, provide standardized benchmarks for comparative analysis. This integration allows for a more standardized and reproducible evaluation of body representation impairments, supporting both clinical and research applications. Future studies should validate the proposed metrics through large-scale datasets, ensuring their clinical relevance and robustness in detecting neuropsychological deficits. Moreover, incorporating digital drawing tools and AI-driven assessment methods could enhance precision and enable real-time monitoring of rehabilitation progress. By structuring qualitative parameters into measurable quantitative indices, this approach offers a foundation for the development of AI models that can assist clinicians in diagnosing and monitoring body representation disturbances in stroke patients. This step represents an essential advancement toward a more objective and scalable assessment of neuropsychological conditions through human figure-drawing analysis.

### 4.7. Limitation

One relevant aspect to highlight concerns the limited number of studies included in the review. In fact, only 10 articles were retrieved. This could be due to the particularly stringent selection criteria that were adopted. Specifically, only studies conducted on poststroke adult populations that used specific graphical representation tools of the human figure (such as the Human Figure Drawing Test, the Draw a Man Test, or the self-portrait) in relation to the construct of body representation were considered. The decision to exclude studies that used pictorial or artistic representations further restricted the number of available studies, but was necessary to ensure methodological homogeneity and focus on standardized or semi-standardized instruments. A further limitation is the high heterogeneity of the selected studies. The included articles differed significantly from each other in terms of experimental design, specific objectives, and mode of application of the graphical representation tool. An additional constraint is found in the different instruments used, the different administration instructions given to patients at the time of administration, the different parameters chosen, and consequently, the different interpretations given to the designs. This variability made direct comparison of the results difficult, but it reflects the variety of experimental approaches in the literature on this topic, highlighting an area of research still under development.

## 5. Conclusions

The use of human figure drawing has been applied in various ways (assessment, prognosis, monitoring of the patient) and in association with different topics, within the context of body representation following a stroke. The strength of these tools lies in their flexibility and versatility, which allow for capturing different aspects of the patient’s functioning, coupled with their ease of administration in diverse contexts. Notably, self-portraits and body drawings emerge as particularly valuable tools for analyzing alterations in body representation post-stroke. However, their interpretation requires careful consideration of cognitive and affective factors, as well as the etiology of brain damage, to ensure a nuanced understanding of the patient’s experience. At the same time, there is a clear need for synthesis and standardization across the different methods of administration and usage to enable greater generalizability of the results. This heterogeneity, both methodologically and thematically, reflects the inherent complexity of body representation itself. The studies underscore that body representation disruptions after stroke cannot be disentangled from the interplay of cognitive, physical, motor, and emotional domains. The findings highlight the impossibility of drawing clear boundaries between these domains, emphasizing the necessity of holistic assessment strategies that integrate both qualitative and quantitative methods. Such an approach is critical not only for comprehending the multifaceted nature of body representation but also for enhancing functional recovery through targeted interventions.

Further studies on large sample sizes and with a homogeneous methodology are needed to verify the usefulness of human figure drawing in terms of prognosis in recovery after a stroke, or as an outcome measure to assess the efficacy of a treatment. In this scenario, the integration of artificial intelligence (AI) models to analyze both qualitative and quantitative features represents an essential advancement. These tools could enable a more objective, scalable, and reproducible assessment of neuropsychological conditions through human figure drawing analysis, addressing current methodological limitations while preserving the richness of patient-centered insights. Future research should prioritize mixed-method frameworks to bridge qualitative data, such as those derived from self-portraits, with AI-driven quantitative metrics, ensuring a comprehensive evaluation of body representation. This dual focus will advance standardized protocols and foster innovation in clinical practice, ultimately improving rehabilitation outcomes and personalizing recovery pathways.

## Figures and Tables

**Table 1:** Score ranges of the original and modified versions of the NOS.

**Observational Studies (Modified NOS, Maximum 10 Points)**	
**Risk of bias**	**Score**
Low	≥7
Moderate	5–6
High	<5
**Cohort Studies (Original NOS, Maximum 9 Points)**	
**Risk of bias**	**Score**
Low	≥6
Moderate	4–5
High	<4

**Table 2:** Main features and results of the 10 studies reviewed.

Study ID	Participants (Number, Sex, Age, Diagnosis)	Aim of the Study	Measure Assessment of Body Representation	Other Assessment Tools	Description of the Results
Bach, P., et al., 1971 [57]	1° part of the study: Numbers: 45 – 25 right hemiplegia; – 20 left hemiplegia. Age: 61 Diagnosis: Cerebrovascular disease 2° part of the study: Numbers: 61 – 26 right hemiplegia; – 35 left hemiplegia. Age: 62 Diagnosis: Cerebrovascular disease	Evaluate the effectiveness of the “Self-Portrait Method” as a predictive tool for determining whether a hemiplegic patient will gain independence in ADL.	Self-portrait method	House drawing	The study results show a correlation between self-portrait denial and the level of independence in ADL, with differences according to the side of hemiplegia. Among patients with right hemiparesis, those who did not show denial (16 patients) exhibited greater independence. In contrast, the 9 patients who showed denial exhibited less autonomy in daily activities. In patients with left lateral hemiparesis, the 15 patients who did not show denial were mostly independent. In contrast, all 5 patients who showed denial exhibited greater functional impairment.
Newman M., 1972 [54]	Numbers: 39 Sex: 24 M, 15 W Age: 55 Diagnosis: Stroke	Investigate the process of neurological and functional recovery in patients with hemiplegia due to cerebral infarction over a period of at least 20 weeks.	Draw a Man Test	Jigsaw puzzle man, Koh block test	Several aspects of post-stroke recovery are examined in this study. Among the patients included in the study, five showed improvement between weeks 5 and 13 in the Draw a Man Test. In some patients, complete constructive apraxia turned into a unilateral body image deficit, while in others, the neglect of space on the affected side decreased, although hemianopsia did not improve. Only two patients showed changes in their visual fields: in both cases, they changed from a tendency to suppress the visual field of the affected half to an apparently normal visual field. This improvement occurred in the third and fourth patients at weeks 3 and 4, respectively.
Chen-Sea M. J., 2000 [49]	Numbers: 51 – 25 right cerebrovascular accident; – 26 hemorrhage CVAs, infarction. Sex: 38 M Age: 59.41 ± 8.66 years Diagnosis: Right brain stroke Controls: Numbers: 110 Sex: 77 M Age: 56.79 ± 10.98 years	Determine the reliability and validity of a Draw a Man Test in measuring personal neglect in patients with right brain stroke, and develop a scoring method for the Draw a Man Test that would 1. Test the interrater reliability of the method; 2. Differentiate persons with personal neglect from those without; 3. Validate its functional significance.	Draw a Man Test	Klein–Bell ADL Scale	In this study, the Draw a Man Test was validated as an assessment tool for personal neglect. Patients who were found to have personal neglect showed poorer performance in ADL than patients without personal neglect. In particular, the group with personal neglect was significantly worse than the group without personal neglect in somatosensory, motor, and muscle strength. The group with personal neglect showed lower scores than the group without personal neglect in five areas of ADL: the most difficult ADL areas on the Klein–Bell ADL Scale for participants with personal neglect were dressing, elimination, mobility, and bathing.
Chen Sea M. J., 2001 [50]	Number: 46 Sex: 34 M, 12 W Age: / Diagnosis: Right brain stroke (hemorrhage and infarction)	Investigate the impact of unilateral neglect on activities of daily living.	Draw a Man Test	Klein–Bell ADL Scale; Random Chinese World Cancellation Test (RCWCT); Physical Status Measures (Motor status evaluation, sensory evaluation)	In this study, the Draw a Man Test was used as a tool to discriminate patients exhibiting personal neglect. Results showed that patients with personal neglect combined with extrapersonal neglect (group D) were significantly more deficient in ADLs than patients exhibiting only extrapersonal neglect (group B) and patients exhibiting no neglect (group A).
Morin C., et al., 2003 [51]	161 stroke – Left Brain Lesions Number: 75 Sex: 38 M 37 W Age: 52.1 – Right Brain Lesions Number: 86 Sex: 51 M 35 W Age: 55 98 Control Sex: 30 M 68 W Age: 63.3; 49 Spinal, radicular, or limb lesions Sex: 29 M 20 W Age: 53.3 25 Spinal cord lesions 24 Traumatic Limb Lesions	Analyze how recent right and left brain vascular lesions affect the specular image in the self-portrait.	HFD	/	Based on the designs produced and through the analysis of 11 parameters, patients were divided into three clusters: Cluster 1: consisting mainly of healthy participants; Cluster 2: characterized by an overrepresentation of patients with speech disorders, particularly aphasics; Cluster 3: consisting almost exclusively of patients with brain lesions in the right hemisphere. Compared with the other two groups, these patients show graphic behaviors or make comments that differ significantly from those of the other participants, suggesting a different way of processing and representing the body.
Morioka S., et al., 2005 [52]	Numbers: 6 Sex: 4 M, 2 W Age: 70.5 Diagnosis: Parietal lobe damage	Examine the left-side neglect tendency in unilateral spatial neglect (USN) patients by assessing their ability to draw self-portraits with their eyes open and closed.	Self-portrait	/	Self-portraits of six patients with unilateral spatial neglect were analyzed with eyes open and eyes closed. With eyes open, all six patients were able to draw their self-portraits, which clearly showed the defects on the left side of the body. However, with their eyes closed, only three patients were able to complete the drawing, while the other three could not. In addition, the self-portraits drawn with eyes closed had more defects than those drawn with eyes open. According to the authors, these results suggest that patients with unilateral spatial neglect not only have difficulty in memorizing the visual scene but also in remembering the mental image of their own bodies.
Corallo, F., et al., 2021 [53]	Number: 46 Sex: 26 M, 20 W Age: 55.33 Diagnosis: Acquired brain injury (traumatic and vascular groups)	Evaluate the existence of correlations between body image and levels of anxiety, depression, and cognitive impairments in ABI patients, assessing differences in outcomes by brain damage etiology.	Body Image Scale (BIS), Human Figure Drawing (HFD)	Montreal Cognitive Assessment (MoCA); Beck’s Depression Inventory (BDI-II); Hamilton Rating Scale for Anxiety (HAM-A); Clinical Insight Rating Scale (CIRS)	In this study, patients are classified as “traumatic” or “vascular” according to the etiology of their brain injury. Higher levels of body image distortion, associated with greater depressive and anxiety symptoms, are observed in the traumatic group. In both the traumatic and vascular groups, the results suggest that better overall cognitive function is associated with a more detailed and accurate representation of the human figure. In addition, the visuospatial subscore of the MoCA emerged as a significant predictor of performance on the HFD test in both groups, highlighting the importance of visuospatial skills in human figure drawing. In the vascular group, other cognitive skills, such as naming, language, abstraction, and orientation, were found to be predictive of HFD performance, suggesting that multiple cognitive domains may influence the ability to graphically represent one’s body.
Caimmi M. et al., 2022 [55]	Numbers: 19 Sex: M: 13, W: 6 Age: 62 ± 9 Diagnosis: Stroke	Test the immediate and sustained effects of the intervention in reducing impairment in chronic stroke and preliminarily verify the effects on activity; regarding body image effects, as well as pre- and post-robotic rehabilitation intervention.	Draw a Person test	Fugl–Meyer Assessment (FMA), Wolf Motor Function Test Time (WMFT TIME), Functional Ability Scale (WMFT FAS, Motor Activity Log, Quality of Movement (MAL QOM), Amount Of Use (MAL AOU)	The Draw a Person test showed heterogeneous results at T0. Two-thirds of the patients’ drawings showed at least one abnormality (the most common: missing or disproportionate body segments and missing facial elements. The results indicated improvements in post-treatment assessments. In particular, the drawn figures appeared more complete, including all body segments with correct proportions. In addition, compared with the baseline assessment, facial features such as eyes, nose, and mouth were added.
Lo Buono V., et al., 2023 [31]	Number: 46 Sex: 26 M, 20 W Age: 55.5 Diagnosis: Acquired brain injury	Evaluate the relationship of mood disorders and body perception in ABI patients.	Body Image Scale (BIS), Human Figure Drawing (HFD)	Montreal Cognitive Assessment (MoCA), Beck Depression Inventory II (BDI-II), Hamilton Rating Scale for Anxiety (HAM-A)	In patients with right hemisphere brain lesions, depression (BDI-II)was found to be a significant predictor of body image perception (BIS), while global cognitive ability (MoCA) predicted the quality of human figure representation in the HFD. In patients with left hemisphere brain lesions, on the other hand, anxiety levels (HAM-A) were a significant predictor for both BIS and HFD. Finally, in patients with bilateral brain lesions, depression (BDI-II) emerged as a significant predictor of human figure drawing quality (HFD), while global cognitive abilities (MoCA) were predictive of both body image perception (BIS) and human figure representation (HFD).
Martinelli I., et al., 2023 [56]	Number: 56 Sex/ Age/ Diagnosis: Chronic stroke (presence of unilateral sensorimotor deficits of the upper limb) 46 Controls	Present and validate the use of a new app called QDraw for the quantitative analysis of drawings and to investigate whether this analysis can reveal distortions of BRs in chronic stroke patients	Human Figure Drawings (HFD)	/	In this study, analysis of the drawings revealed a significant difference between stroke patients and healthy subjects, with the latter tending to represent the human figure in more detail. In particular, the drawings of healthy subjects more frequently included elements related to general body appearance (such as hair and clothing), facial features (nose, mouth, eyes, ears), and upper and lower limbs. Regarding the analysis of limb asymmetry, the Wilcoxon test identified a significant difference between arms and legs in the drawings of stroke patients. In particular, the asymmetry index of the upper limb was significantly higher than that of the lower limb. This suggests that, in the patients’ drawings, the asymmetry between the upper limbs was more pronounced than that of the lower limbs.

ADL: Activities of daily living; CVA: cerebrovascular accident; RCWCT: Random Chinese Word Cancelation Test; BIS: Body Image Scale; HFD: Human figure drawing; FMA: Fugl–Meyer Assessment; WMFT: Wolf Motor Function Test; WMFT FAS: Wolf Motor Function Test—Functional Ability Scale; MAL QOM: Motor Activity Log, Quality of Movement; MAL AOU: Motor Activity Log, Amount Of Use; ABI: Acquired brain injury; BDI-II: Beck Depression Inventory II; MoCA: Montreal Cognitive Assessment; HAM-A: Hamilton Rating Scale for Anxiety.

**Table 3:** Characteristics of the graphic human representation instruments.

Study ID	Clinical Use and Topic	Measure Assessment of Body Representation	Parameters Used for Graphical Representation Interpretation	Prevalent Evaluation Type	Test Delivery
Bach, P. et al., 1971 [57]	Prognosis—ADL	Self-portrait method	Presence or absence of body parts (es. face, 4 extremities)	Qualitative	Self-portrait
Newman M., 1972 [54]	Monitoring—Neglect	Draw a Man Test	Qualitative longitudinal analysis of drawings	Qualitative	Not stated
Chen-Sea M. J., 2000 [49]	Prognosis- Neglect—ADL	Draw a Man Test	Unilateral body parts representation	Qualitative	Generic human figure production
Chen Sea M. J., 2001 [50]	Prognosis—Neglect—ADL	Draw a Man Test	Unilateral body parts representation	Qualitative	Not stated
Morin C., et al., 2003 [51]	Patient evaluation	Human figure drawings	Parameters are extracted from the drawings and used in a multivariate analysis: (a) Location on the sheet; (b) Axis of symmetry (vs 20 deg from vertical); (c) Body representation (frontal, 3/4, or profile); (d) Lateral orientation; (e) Graphic type (clothed figure, pictogram or volumes, outline, or naked); (f) Hands (visible, absent with mouth, cut off or stump-like); (g) Feet (visible, cut off, stump-like); (h) Mouth visible; (i) Eyes visible; (j) Adaptive equipment; (k) Hemineglect in portrait.	Quantitative	Self-portrait
Morioka S., et al., 2005 [52]	Patient evaluation—Neglect	Self-portrait	/	Qualitative	Self-portraits, one by keeping eyes opened and one closed
Corallo, F. et al., 2021 [53]	Patient evaluation—Mood	Body Image Scale (BIS), Human Figure Drawing (HFD)	43 details analyzed, but not specified. Generically stated the evaluation of physical characteristics and drawing lines.	Quantitative	Drawing of two human figures of opposite sexes
Caimmi, M. et al., 2022 [55]	Monitoring	Draw a Person test	Qualitative analysis	Qualitative	Not stated
Lo Buono V., et al., 2023 [31]	Patient evaluation—Mood	Body Image Scale (BIS), Human Figure Drawing (HFD)	43 details analyzed, but not specified. Generically stated the evaluation of physical characteristics and drawing lines.	Quantitative	Drawing of two human figures of opposite sexes
Martinelli I., et al., 2023 [56]	Patient evaluation	Human figure drawings	General appearance of the human figure (stick figure, clothes, hair); Face characterization (head, nose, mouth), eyes (both eyes, only one eye, no eye), ears (both ears, only one ear, no ear); Upper body characterization (arms [both arms, both arms connected, only one arm, no arm] hands [both hands, only one hand, no hand]; Lower body characterization (legs [both legs, both legs connected, only one leg, no leg], feet [both feet, only one foot, no foot]); Body segment metrics (lengths, asymmetry index for arms lengths).	Quantitative	Generic human figure production

**Table 4:** Body image representation evaluation parameters.

Qualitative Parameters	Quantitative Parameters
Eyes, ears, feet, clothes, hair, hands	Leg and arm length and width
Limbs attached or detached from the torso	Head size
Body representation (frontal, ¾ representation, profile representation)	Axis of symmetry (<20° to vertical, >20° leftwards, >20° rightwards)
Hemineglect in portrait (unilateral body parts representation)	Location on the sheet (middle third, left third, right third)
Lateral orientation or facing representation	Overall height of the figure
Structural disorganization	Shoulder and hip width
Perseverations	
Simultaneous agnosia	
Overcopying	
Unrelated activity	
Severe denial in self-portraits	
Lack or disproportion of body segments (hands, feet, and entire upper and lower limbs)	

**Table 5:** Risk of bias assessment.

**Cohort Studies—Newcastle–Ottawa Scale (NOS)—max. 9 Points**						
**ID**	**Study**	**Participant Selection**	**Group Comparability**	**Outcome Assessment**	**Total**	**Risk of Bias**
Newman M., et al., 1972 [54]	Cohort	3	0	3	6	Low
Caimmi M., et al., 2022 [55]	Cohort	3	0	1	4	Moderate
Bach P., et al., 1971 [57]	Cohort	3	0	1	4	Moderate
**Cross-Sectional Studies—Modified Newcastle–Ottawa Scale (NOS)—max. 10 Points**						
**ID**	**Study**	**Participant Selection**	**Group Comparability**	**Outcome Assessment**	**Total**	**Risk of Bias**
Corallo F., et al., 2021 [53]	Cross-sectional	4	1	2	7	Low
Lo Buono V., et al., 2023 [31]	Cross-sectional	4	1	2	7	Low
Martinelli I., et al., 2023 [56]	Cross-sectional	4	1	2	7	Low
Chen Sea M. J., 2000 [49]	Cross-sectional	4	1	2	7	Low
Chen Sea M. J., 2001 [50]	Cross-sectional	4	1	2	7	Low
Morin C., et al., 2003 [51]	Cross-sectional	4	1	2	7	Low
Morioka S., et al., 2005 [52]	Cross-sectional	3	0	2	5	Moderate

**Table 6:** Conversion from parameters used to measurements for AI models.

Qualitative Parameter	Quantitative Measure for AI Models
Eyes, ears, feet, clothes, hair, hands	Presence (binary: 0/1), number of features detected, symmetry, size ratios, feature completeness (%)
Limbs attached or detached from torso	Limb connectivity score (0–1), number of disconnected limbs, average limb-torso distance (pixels)
Body representation	Angle of representation (degrees), proportion of body visible (%)
Hemineglect in portrait	Ratio of left vs. right side features detected, asymmetry score (%)
Lateral orientation or facing	Head/body orientation angle (degrees), eye gaze direction
Structural disorganization	Deviation from expected body structure (%), intersection of limbs, misplaced features count
Perseverations	Number of redundant strokes, stroke overlap percentage, pattern repetition detection
Simultaneous agnosia	Object completeness score, missing elements count, complexity reduction (%)
Overcopying	Extra elements detected beyond expected count, stroke duplication ratio
Unrelated activity	Presence of extraneous objects, number of unrelated features detected, semantic consistency score
Leg and arm length and width	Absolute length/width (pixels, cm), proportion to torso size, left-right symmetry (%)
Head size	Ratio to body size, absolute head height, and width (pixels, cm), proportionality index (%)
Axis of symmetry	Deviation from vertical (°), classified as <20° (aligned), >20° leftwards, >20° rightwards
Location on the sheet	Position classification: middle third, left third, right third
Overall height of the figure	Absolute height (pixels, cm), proportion to paper size (%)
Shoulder and hip width	Absolute width (pixels, cm), proportion to body size, left-right symmetry (%)

## Data Availability

The original contributions presented in this study are included in the article/Supplementary Materials. Further inquiries can be directed to the corresponding author.

## Supplementary Materials

The following supporting information can be downloaded at https://www.mdpi.com/article/10.3390/jcm14093098/s1, Table S1: Keywords and the MeSH terms used to formulate the search strategy for PubMed, Scopus, Embase, and Web of Science; Table S2: Search string used for Google Scholar.; PRISMA_2020_checklist; PRISMA_2020_abstract_checklist.

## Abbreviations

The following abbreviations are used in this manuscript:

DAPT	Draw a Person Test
HFD	Human Figure Drawings
USN	Unilateral spatial neglect
ADL	Activities of daily living
PRISMA	Preferred Reporting Items for Systematic Reviews and Meta-Analysis
NOS	The Newcastle-Ottawa Scale
ABI	Acquired Brain Injury
CVA	Cerebrovascular accident
RCWCT	Random Chinese Word Cancelation Test
BIS	Body Image Scale
FMA	Fugl–Meyer Assessment
WMFT	Wolf Motor Function Test
WMFT FAS	Wolf Motor Function Test—Functional Ability Scale
MAL QOM	Motor Activity Log, Quality of Movement
MAL AOU	Motor Activity Log, Amount Of Use
BDI-II	Beck Depression Inventory II
MoCA	Montreal Cognitive Assessment
HAM-A	Hamilton Rating Scale for Anxiety
